# Does Reducing Alcohol Use Among People with HIV Alleviate Psychological Distress and Symptoms of Depression? A Randomized Controlled Trial in Tshwane, South Africa

**DOI:** 10.1007/s10461-023-04205-x

**Published:** 2023-10-19

**Authors:** Jason Bantjes, Neo K. Morojele, Bronwyn Myers, Sonja Swanevelder, Charles Parry

**Affiliations:** 1 Mental health, Alcohol, Substance use and Tobacco (MAST) Research Unit, South African Medical Research Council, Cape Town, South Africa; 2 Department of Psychiatry and Mental Health, University of Cape Town, Cape Town, South Africa; 3 Department of Psychology, University of Johannesburg, Johannesburg, South Africa; 4 Curtin enAble Institute, Curtin University, Perth, Australia; 5 Biostatistics Unit, South African Medical Research Council, Cape Town, South Africa; 6 Department of Psychiatry, Faculty of Medicine and Health Sciences, Stellenbosch University, Stellenbosch, South Africa

**Keywords:** Depression, Alcohol use, HIV, Randomised controlled trial, South Africa

## Abstract

Although alcohol use is associated with depression, it is unclear if brief alcohol reduction interventions can ameliorate depression and psychological distress among people with HIV (PWH). We use data from a two-arm randomised controlled trial to examine this question. PWH on antiretroviral treatment (ART) were randomly assigned to receive a brief intervention or treatment as usual (n = 622). Screening was done with the Alcohol Use Disorders Identification Test (AUDIT), AUDIT-C, Centre for Epidemiological Studies Depression inventory and Kessler Psychological Distress Scale, at baseline and at 3- and 6-months post-baseline. Changes in depression and psychological distress was assessed using analysis of covariance models with baseline measures of alcohol consumption, sex and age included as covariates and adjusting for baseline symptom severity. Changes in alcohol consumption between baseline and follow-up were included in the analysis to establish if this affected outcomes. For both the intervention and control groups, there were significant reductions in symptom severity at 3-months and 6-months for depression and psychological distress, but no significant between group differences were observed. Reductions in alcohol consumption were significantly associated with reductions in depression and psychological distress, supporting the hypothesis that alcohol use is linked to depression among PWH.

*Trial Registration* Pan African Clinical Trials Register, PACTR201405000815100.nh

## Introduction

People with HIV (PWH) are vulnerable to depression, alcohol use disorder (AUD), and psychological distress [1–3]. There is increasing awareness of the need for transdiagnostic interventions for common mental disorders that can be integrated into routine HIV treatment [4]. Systematic reviews of the prevalence of depression, AUD and psychological distress among PWH globally have yielded prevalence estimates of 31% (95% CI 8–34) [5], 29.8% (95% CI 24.1–35.7) [6], and 43.7% (95% CI 29.9–57.5) [7], respectively. Depression and AUDs have a negative impact on antiretroviral therapy (ART) adherence and HIV treatment outcomes, highlighting the need for scalable evidence-based interventions to treat depression and alcohol use among PWH [8–10], particularly in resource constrained environments like South Africa (SA) which has one of the highest rates of HIV infection in the world [11].

Alcohol use and depression are strongly associated, with the presence of either disorder doubling the risk of the other [12, 13]. These associations cannot be accounted for fully by common factors that influence both conditions. Rather these conditions appear to be causally linked. Although the causal relationship is bidirectional, there is evidence from longitudinal studies showing that alcohol use leads to and maintains depression through neurophysiological, metabolic and circadian rhythm changes resulting from exposure to alcohol [13]. Longitudinal studies suggest that reductions in alcohol use ameliorate symptoms of depression among people living with and without HIV [14]. Furthermore, convincing evidence shows that the course of harmful alcohol use can be effectively altered by brief interventions, which can be feasibly implemented in primary health care settings [15–17]. One systematic review and meta-analysis found that interventions targeting excessive alcohol use in young people resulted in a significant reduction in depression symptoms [18], but it is unclear if this is also the case among PWH. Our study aimed to establish whether a brief intervention focused on reducing alcohol consumption among PWH who met criteria for hazardous alcohol use but not dependence has any significant effect on symptoms of depression among patients on antiretroviral therapy in Tshwane, SA.

## Methods

### Design

We conducted a two-arm randomised controlled trial (RCT) with PWH (n = 622) recruited from six ART clinics at public hospitals in Tshwane, SA. Trial methods are fully described in Parry et al. [19].

### Procedure

Patients receiving ART were screened for hazardous alcohol use using the Alcohol Use Disorders Identification Test (AUDIT) and AUDIT-C [20]. Patients ≥ 18 years old were invited to participate in the study if they: (a) were on ART for HIV for at least 3 months and were not being treated for tuberculosis; (b) met criteria for current (past year) harmful/hazardous drinking (AUDIT-C score ≥ 4 for men and ≥ 3 for women), but not for alcohol dependence (total AUDIT score < 23 out of possible 40); (c) were resident in/around Tshwane Metro and not enrolled in another trial; and (d) did not have an extremely poor general health/functional status (Karnofsky clinical score > 50) [21]. Recruitment took place between May 2016 and October 2017. Baseline assessments were done prior to randomisation, and follow-up assessments were conducted at 3-months and 6-months post baseline by research staff who were blind to assignment. Participants received grocery vouchers (valued at ZAR 80 for initial visits and ZAR 100 for follow-up assessments) to reimburse them for their time and were also reimbursed for their transport costs (ZAR 50 per visit).

### Details of the Intervention

Participants assigned to the intervention arm received a manualised intervention comprising four modules of motivational interviewing and problem-solving therapy, delivered in two contact sessions over a period of 4 weeks by project staff with previous counselling training [19]. In two prior RCTs the intervention content was shown to reduce significantly hazardous alcohol and other substance use as well as depression [22, 23]. However, the version of the intervention used in the current study differs from previous versions in that it focuses only on alcohol use and is delivered in 2 contact sessions; previously the content included training on regulating negative emotions and rumination, and was delivered respectively in four and five contact sessions over 4 weeks [22, 23]. The intervention consisted of motivational interviewing (MI) and problem-solving therapy (PST), a form of cognitive behavioural therapy, both of which are well established brief interventions for reducing alcohol consumption particularly when alcohol is used as a strategy for coping with negative emotions and life problems [24]. The intervention focused on helping participants learn and practice adaptive problem solving skills, strategies to identify and regulate uncomfortable emotions, and skills to sustain alcohol behaviour change.

The staff who delivered the intervention received 40 h of intervention-specific training by the psychologists who developed the intervention, as well as ongoing clinical supervision to support them with the intervention. Participants received a workbook which summarised the key content of the intervention and included homework exercises. All modules included opportunities to apply newly learned skills through activities. Participants in the control arm received treatment as usual (TAU), which consisted of the standard package of care for PWH who report hazardous/harmful alcohol use in public health clinics. Specifically, patients who are suspected of having alcohol use problems are referred to on-site psychologists or social workers, if available, or off-site community mental health or alcohol services.

### Data Collection

Basic sociodemographic data (i.e., sex, age, and length of time on ART) and data about alcohol consumption were collected upon enrolment in the study. Alcohol consumption was assessed using the AUDIT and AUDIT-C. The Centre for Epidemiological Studies Depression inventory (CESD-10) and the Kessler Psychological Distress Scale (K10) were used to assess depressive symptoms and levels of psychological distress respectively [25, 26], at baseline and then again at 3- and 6-months post-baseline. The CES-D-10 is a ten-item self-report questionnaire that measures symptoms of depression on a four-point Likert scale, with three items assessing depressed affect, five items assessing somatic symptoms, and two assessing positive affect. Total scores range from 0 to 30, with a score of 10 or more indicating clinically significant symptoms. [25]. The CES-D-10 has good psychometric properties and has been widely used in various cultural contexts and with various clinical populations, including in SA [27, 28] and among PWH [29, 30]. Furthermore, the instrument has adequate sensitivity and specificity for identifying individuals who are likely to meet DSM criteria for a depressive disorder [31, 32]. The K10 is a ten-item self-report questionnaire that assesses common symptoms of psychological distress on a scale from 10 to 50. The instrument is widely used to identify individuals who are likely to meet criteria for a psychiatric disorder and as a measure of outcomes following treatment for common mental disorders. Individuals who score less than 20 on the K10 are likely to be well, those who score between 20 and 29 are likely to have a mild to moderate mental disorder, while those scoring 30 and over are likely to have a severe mental disorder. The K10 has robust psychometric properties for the detection of distress in the general population [26]. The instrument has been used in outpatient settings in SA and showed good construct validity and reliability, with a Cronbach’s alpha of 0.84 and omega total of 0.88 [33].

### Data Analysis

Sample characteristics and changes in aggregate measures of depression and psychological distress between baseline and follow-up for both the intervention and control groups are reported using descriptive statistics. Multiple imputation was used to impute missing data prior to substantive analysis. We used an intention-to-treat analysis to investigate changes in symptoms of depression and levels of psychological distress. The effectiveness of the intervention for reducing depression (measured by the CESD-10) and psychological distress (measured by K10) was assessed using repeated general linear models to assess changes in depression symptoms and psychological distress by sex and age, controlling for baseline measures of alcohol consumption and baseline symptom severity. Group differences were evaluated using analysis of covariance (ANCOVA) models with the baseline measures of symptom severity, sex and age included as covariates and adjusting for baseline alcohol consumption and symptom severity. In these models we also included changes in level of alcohol consumption between baseline and both follow-up points, to investigate if changes in alcohol consumption were associated with changes in depression and psychological distress at both follow-ups. The models included a repeated statement and had an uncorrelated covariance structure. Alpha was set to 0.05 for all tests of significance, unless stated otherwise.

## Results

### Sample Characteristics

We recruited 622 participants who were randomly assigned to either the intervention group (n = 302) or control group (n = 318). Sample characteristics for the total sample and each of the intervention arms are shown in Table 1. The mean age of the participants was 41.0 years (SD = 9.0) and 57.5% self-identified as women. The mean AUDIT score at baseline was 9.5 (SD = 4.7), while the mean CESD-10 and K10 scores were 9.5 (SD = 6.5) and 18.8 (SD = 8.2), respectively. The intervention and control groups did not differ significantly from each other in terms of age, or scores on the AUDIT, CESD-10, and K-10 (p > 0.05), although there were marginally more females in the intervention group (53.1% vs. 46.9%, χ2(2) = 5.706, p = 0.017). Between the two sexes, there were no significant differences in age or baseline depression scores, although baseline levels of psychological distress did differ significantly, with K10 scores marginally higher among women (mean = 19.7, SD = 8.6) compared to men (mean = 17.6, SD = 7.4).

**Table 1:** Sample characteristics for total sample and each of the intervention arms at baseline

	Total sample (n = 622)		Control group (n = 318)		Treatment group (n = 304)			p
Gender (female)	57.5%		46.9%		53.1%		X2(2) = 5.706	0.017*
Mean age (SD)	41.0 (9.0)		41.96 (9.1)		39.91 (8.9)		F = 0.075	0.785
Mean CESD-10 score	9.5 (6.5)		9.23 (6.8)		9.83 (6.3)		F = 1.440	0.231
Mean K-10 score	18.8 (8.2)		18.39 (8.2)		19.21 (8.1)		F = 0.007	0.931
Mean AUDIT total score	9.5 (4.7)		9.65 (4.6)		9.39 (4.7)		F = 0.000	0.983

*p < 0.05

### Aggregate Intervention Effects

The mean symptoms scores for depression and psychological distress at baseline and follow-up for the treatment and control groups are shown in Table 2. Reductions in symptoms of depression and levels of psychological distress were observed in both groups. However, no significant differences in mean symptoms scores for depression or psychological distress were observed between the intervention and control groups at baseline or at follow-up (p > 0.05).

**Table 2:** Mean symptoms scores for depression and psychological distress at baseline and 3-month and 6-month follow-up for treatment and control group

	Baseline				3-month follow-up				6-month follow-up			
	Mean	SD	t	p	Mean	SD	t	p	Mean	SD	t	p
Symptoms of depression												
Treatment group	9.83	6.325	− 1.141	0.127	8.62	6.506	− 0.634	0.263	8.02	6.275	− 1.066	0.143
Control group	9.23	6.754			8.25	6.402			7.43	6.502		
K10 total score												
Treatment group	19.21	8.116	− 1.143	0.127	18.2	8.242	− 0.348	0.364	16.9	7.375	0.278	0.39
Control group	18.39	8.204			17.76	7.611			16.81	7.901		

The proportion of participants with clinically significant symptoms of depression (defined as a score of 10 or more on the CESD-10), as well as mild, moderate, and severe symptoms of psychological distress are shown in Table 3. High rates of clinically significant depression symptoms and psychological distress were observed in both groups at baseline which declined over time, although there were no statistically significant differences between the control and intervention group at follow-up (p > 0.05).

**Table 3:** Proportion of participants with clinically significant depression scores and levels of psychological distress (mild, moderate, and severe), at baseline and 3-month and 6-month follow-up for treatment and control groups

	Baseline		3MFU		6MFU		X2(2)	p
	n	%	n	%	n	%		
Depression								
CESD-10 > 10								
Control group	145	45.60	108	39.60	92	32.90	0.92392	0.630
Treatment group	144	47.40	91	41.60	91	36.00		
Psychological distress								
None								
Control group	208	65.4	185	68.0	203	72.5	0.715	0.699
Treatment group	184	60.3	147	66.2	181	71.3		
Mild/moderate								
Control group	72	22.6	62	22.8	54	19.3	1.819	0.403
Treatment group	87	28.5	54	24.3	59	23.2		
Severe								
Control group	38	11.9	25	9.2	23	8.2	0.906	0.636
Treatment group	34	11.1	21	9.5	14	5.5		

### Rates of Remission and Deterioration

The rates of remission for depressive symptoms (i.e., the proportion of participants who moved from having clinically significant symptoms at baseline to having clinically insignificant symptoms at follow-up) and rates of deterioration (i.e., the proportion of participants who moved from having clinically insignificant symptoms at baseline to reporting clinically significant symptoms at follow-up) in the control and intervention groups are presented in Table 4. No significant differences in rates of remission for depression were observed between control group and treatment group at 3-month (17.0% vs. 20.0%, t = − 0.92, p = 0.18) or 6-month follow-up (25.0% vs. 25.0%, t = − 0.07. p = 0.47). Similarly, no significant differences in deterioration rates between control group and treatment group were observed at 3-month (12.0% vs. 13.0%, t = − 0.23, p = 0.41) or 6-month follow-up (11.0% vs. 14.0%, t = − 1.2, p = 0.11).

**Table 4:** Rates of remission^a^ and deterioration^b^ in clinically significant depressive symptoms for control and intervention groups, at 3-month and 6-month follow-up

	N	Mean (%)	S. E (%)	t	p
Remission^a^					
3-month follow-up					
Control group	46	17.0	2.3	− 0.92	0.18
Treatment group	44	20.0	2.7		
6-month follow-up					
Control group	69	25.0	2.6	− 0.07	0.47
Treatment group	63	25.0	2.7		
Deterioration^b^					
3-month follow-up					
Control group	33	12.0	2.0	− 0.23	0.41
Treatment group	28	13.0	2.3		
6-month follow-up					
Control group	30	11.0	1.9	− 1.20	0.11
Treatment group	36	14.0	2.2		

^a^Remission is defined as a change in CESD-10 from above 10 to below 10

^b^Deterioration is defined as a change in CSED-10 score from below 10 to above 10

### Intervention Effectiveness

Intervention effectiveness was assessed with an analysis of covariance (ANCOVA), controlling for baseline symptoms severity and level of alcohol consumption, and including sex, age and change in alcohol consumption as co-variates. In the ANCOVA model, age had no effect on outcome for depression F(1,457) = 1.42, p = 0.234) or psychological distress F(1,470) = 1.85, p = 0.175), and was removed from the final model. For both the intervention and control groups, there were significant reductions in symptom severity at 3-months and 6-months for depression F(1,443) = 4.05, p = 0.0448) and psychological distress F(1,448) = 9.89, p = 0.002). However, there were no significant between group differences at 3-months or 6-months follow-up for depression F(1,443) = 0.01, p = 0.907) or psychological distress F(1,448) = 0, p = 0.968). Baseline symptoms severity had a significant effect on symptoms scores at follow-up for both depression F(1,449) = 116.59, p ≤ 0.0001) and psychological distress F(1, 461) = 144.53, p < 0.0001). Significant differences were observed between sexes, for depression (F(1,460) = 8.29, p = 0.004) and psychological distress (F(1,466) = 10.45, p < 0.001), with better treatment responses observed among men. Finally, reductions in depression scores were significantly associated with reductions in alcohol consumption at 3-months (F(1,434) = 5.36, p = 0.021) and 6-months (F(1,462) = 5.67, p = 0.018). Reductions in levels of psychological distress were also significantly associated with reductions in alcohol consumption at 3-month (F(1,446) = 6.46, p = 0.011) and 6-month (F(1,473) = 7.96, p = 0.005) follow-up.

## Discussion

The results of this RCT demonstrate that a brief intervention focused on reducing alcohol consumption among PWH who report problem drinking (but not dependence) can result in significant reductions in symptoms of depression and levels of psychological distress at both 3- and 6-months post-intervention, irrespective of the severity of the problem drinking or symptom severity at baseline. However, the symptom reductions observed in the intervention group did not differ significantly from those in the TAU group. Furthermore, our data show that reductions in alcohol consumption are significantly associated with reductions in depression and psychological distress. Our study illustrates how brief interventions for emotional and behavioural problems when delivered by trained research staff under supervision, can be successfully delivered in HIV treatment centres, which is consistent with previous studies showing that brief interventions can be effectively delivered in primary health care settings [15, 16]. It will be important for subsequent studies to establish if the brief intervention we have tested here has the same outcomes when delivered by usual staff in the context of usual care in these settings.

In this RCT our structured brief intervention was no more effective than TAU, which consists of unstructured counselling and referrals to trained mental health professionals for psychotherapy. Previously, in two separate studies (i.e. projects STRIVE and MIND), our brief intervention was shown to be more effective than TAU [22, 23], although for project STRIVE the intervention was delivered over five sessions a week apart (so there was more time for people to engage with the content and make changes.) and in MIND the intervention was delivered over 4 sessions a week apart and included content directly focused on managing negative emotions and rumination. It is possible that in the current trial, delivering the intervention in only 2 contact sessions reduced the effectiveness of the intervention compared to TAU, suggesting that future studies should investigate the dose effect of the intervention.

Our finding of significant reductions in depression and psychological distress, following an intervention aimed narrowly at reducing alcohol consumption, are congruent with the results of previous longitudinal studies showing that reductions in alcohol consumption led to reductions in depressive symptoms [12, 18]. Crucially, our data demonstrate that changes in levels of alcohol consumption are associated with reductions in depressive symptoms, supporting the hypothesis that problem drinking precipitates and/or maintains depression and psychological distress [14].

Finally, the results of this study are a good example of the potential for transdiagnostic interventions that simultaneously effect changes in symptoms of multiple disorders, by addressing common underlying mechanisms that precipitate and maintain these disorders.

This study has two important limitations. Firstly, although we used well-validated screening instruments to assess alcohol use, depression and psychological distress, the study would have been even more convincing if participants had been assessed by trained mental health professionals using structured clinical interviews. Secondly, although the study was sufficiently powered to assess changes in the outcome measures, the sample was not large enough for a more sophisticated analysis of baseline predictors of treatment outcome. It would be helpful if future studies in this area were able to move towards a precision medicine approach by developing prediction algorithms to identify PWH who are likely to benefit most from brief structured interventions like ours to reduce alcohol consumption. Reliable methods for assigning PWH to the treatments they are most likely to benefit from is particularly important in resource constrained environments, like SA, where scarce resources need to be allocated to patients who are most likely to benefit from them.

## Conclusion

Our study illustrates how brief interventions to reduce alcohol consumption can be effectively delivered in primary health care settings, such as HIV treatment centres, in resource constrained environments. Crucially, this study provides evidence of how brief interventions to reduce alcohol consumption among PWA can significantly ameliorate symptoms of depression and psychological distress, providing further evidence to support the hypothesis that alcohol use is causally linked to depressive disorders.

## Data Availability

With written request and after review and approval by the Principal Investigators (CP and NKM), a data dictionary defining each field in the analytic data set and de-identified individual participant data will be made available after findings of the main analyses have been published.

## References

[1] Brandt R (2009). The mental health of people living with HIV/AIDS in Africa: a systematic review. Afr J AIDS Res.

[2] O’Cleirigh C, Magidson JF, Skeer MR, Mayer KH, Safren SA (2015). Prevalence of psychiatric and substance abuse symptomatology among HIV-infected gay and bisexual men in HIV primary care. Psychosomatics.

[3] Kagee A, Saal W, Bantjes J, Sterley A (2021). Correlates of viral non-suppression among South African antiretroviral therapy users: comorbidity of major depression, posttraumatic stress, and alcohol use disorders. AIDS Care.

[4] Chibanda D, Cowan FM, Healy JL, Abas M, Lund C (2015). Psychological interventions for common mental disorders for people living with HIV in low- and middle-income countries: systematic review. Trop Med Int Health.

[5] Rezaei S, Ahmadi S, Rahmati J, Hosseinifard H, Dehnad A, Aryankhesal A (2019). Global prevalence of depression in HIV/AIDS: a systematic review and meta-analysis. BMJ Support Palliat Care.

[6] Duko B, Ayalew M, Ayano G (2019). The prevalence of alcohol use disorders among people living with HIV/AIDS: a systematic review and meta-analysis. Subst Abuse Treat Prev Policy.

[7] Ma H, Zhu F, Zhai H, Ma Y, Liu Y, Wang S (2022). Prevalence of psychological distress among people living with HIV/AIDS: a systematic review and meta-analysis. AIDS Care.

[8] Azar MM, Springer SA, Meyer JP, Altice FL (2010). A systematic review of the impact of alcohol use disorders on HIV treatment outcomes, adherence to antiretroviral therapy and health care utilization. Drug Alcohol Depend.

[9] Nakimuli-Mpungu E, Bass JK, Alexandre P, Mills EJ, Musisi S, Ram M (2012). Depression, alcohol use and adherence to antiretroviral therapy in sub-Saharan Africa: a systematic review. AIDS Behav.

[10] Mayston R, Kinyanda E, Chishinga N, Prince M, Patel V. Mental disorder and the outcome of HIV/AIDS in low-income and middle-income countries: a systematic review. AIDS [Internet]. 2012 [cited 2022 Oct 24];26. Available from: https://journals.lww.com/aidsonline/Fulltext/2012/12002/Mental_disorder_and_the_outcome_of_HIV_AIDS_in.4.aspx. 10.1097/QAD.0b013e32835bde0f 23303434

[11] South African National HIV, Prevalence I, Behaviour and, Survey C. 2017: towards achieving the UNAIDS 90-90-90 targets. 2019 [cited 2022 Dec 8]; Available from: https://repository.hsrc.ac.za/handle/20.500.11910/15052.

[12] Boden JM, Fergusson DM (2011). Alcohol and depression. Addiction.

[13] McHugh R, Weiss RD (2019). Alcohol use disorder and depressive disorders. Alcohol Res..

[14] Sullivan LE, Goulet JL, Justice AC, Fiellin DA (2011). Alcohol consumption and depressive symptoms over time: a longitudinal study of patients with and without HIV infection. Drug Alcohol Depend.

[15] Moyer A, Finney JW, Swearingen CE, Vergun P (2002). Brief interventions for alcohol problems: a meta-analytic review of controlled investigations in treatment-seeking and non-treatment-seeking populations. Addiction.

[16] Bien TH, Miller WR, Tonigan JS (1993). Brief interventions for alcohol problems: a review. Addiction.

[17] van der Westhuizen C, Myers B, Malan M, Naledi T, Roelofse M, Stein DJ (2019). Implementation of a screening, brief intervention and referral to treatment programme for risky substance use in South African emergency centres: a mixed methods evaluation study. PLoS ONE..

[18] Fredman Stein K, Allen JL, Robinson R, Smith C, Sawyer K, Taylor G (2022). Do interventions principally targeting excessive alcohol use in young people improve depression symptoms? A systematic review and meta-analysis. BMC Psychiatry.

[19] Parry CDH, Morojele NK, Myers BJ, Kekwaletswe CT, Manda SOM, Sorsdahl K (2014). Efficacy of an alcohol-focused intervention for improving adherence to antiretroviral therapy (ART) and HIV treatment outcomes—a randomised controlled trial protocol. BMC Infect Dis.

[20] Morojele NK, Nkosi S, Kekwaletswe CT, Shuper PA, Manda SO, Myers B (2016). Utility of Brief Versions of the Alcohol Use Disorders Identification Test (AUDIT) to identify excessive drinking among patients in HIV care in South Africa. J Stud Alcohol Drugs.

[21] Karnofsky D. The clinical evaluation of chemotherapeutic agents in cancer. Evaluation of chemotherapeutic agents [Internet]. 1949 [cited 2022 Dec 8]; Available from: https://cir.nii.ac.jp/crid/1570291225027627008.

[22] Myers B, Lombard CJ, Lund C, Joska JA, Levitt N, Naledi T (2022). Comparing dedicated and designated approaches to integrating task-shared psychological interventions into chronic disease care in South Africa: a three-arm, cluster randomised, multicentre, open-label trial. The Lancet.

[23] Sorsdahl K, Stein DJ, Corrigall J, Cuijpers P, Smits N, Naledi T (2015). The efficacy of a blended motivational interviewing and problem solving therapy intervention to reduce substance use among patients presenting for emergency services in South Africa: a randomized controlled trial. Subst Abuse Treat Prev Policy.

[24] Ghosh A, Singh P, Das N, Pandit PM, Das S, Sarkar S (2022). Efficacy of brief intervention for harmful and hazardous alcohol use: a systematic review and meta-analysis of studies from low middle-income countries. Addiction.

[25] Andresen EM, Malmgren JA, Carter WB, Patrick DL (1994). Screening for depression in well older adults: evaluation of a short form of the CES-D. Am J Prev Med.

[26] da Silva BFP, Santos-Vitti L, Faro A (2021). Kessler Psychological Distress Scale: internal structure and relation to other variables. Psico-USF.

[27] Kilburn K, Prencipe L, Hjelm L, Peterman A, Handa S, Palermo T (2018). Examination of performance of the Center for Epidemiologic Studies Depression Scale Short Form 10 among African youth in poor, rural households. BMC Psychiatry.

[28] Baron EC, Davies T, Lund C (2017). Validation of the 10-item Centre for Epidemiological Studies Depression Scale (CES-D-10) in Zulu, Xhosa and Afrikaans populations in South Africa. BMC Psychiatry.

[29] Mueses-Marín HF, Montaño D, Galindo J, Alvarado-Llano B, Martínez-Cajas J (2019). Psychometric properties and validity of the Center for Epidemiological Studies Depression Scale (CES-D) in a population attending an HIV clinic in Cali, Colombia. Biomedica.

[30] Zhang W, O’Brien N, Forrest JI, Salters KA, Patterson TL, Montaner JSG (2012). Validating a Shortened Depression Scale (10 Item CES-D) among HIV-positive people in British Columbia, Canada. PLoS ONE.

[31] Irwin M, Artin KH, Oxman MN (1999). Screening for depression in the older adult: criterion validity of the 10-item Center for Epidemiological Studies Depression Scale (CES-D). Arch Intern Med.

[32] Nishiyama T, Ozaki N, Iwata N (2009). Practice-based depression screening for psychiatry outpatients: feasibility comparison of two-types of Center for Epidemiologic Studies Depression Scales. Psychiatry Clin Neurosci.

[33] Hoffman J, Cossie Q, Ametaj AA, Kim HH, James R, Stroud RE (2022). Construct validity and factor structure of the Kessler-10 in South Africa. BMC Psychol.

